# Chronic Temporomandibular Disorders: disability, pain intensity and fear of movement

**DOI:** 10.1186/s10194-016-0690-1

**Published:** 2016-11-03

**Authors:** Alfonso Gil-Martínez, Mónica Grande-Alonso, Ibai López-de-Uralde-Villanueva, Almudena López-López, Josué Fernández-Carnero, Roy La Touche

**Affiliations:** 1 Hospital La Paz Institute for Health Research, Madrid, Spain; 2 Motion in Brains Research Group, Departamento de Fisioterapia, Centro Superior de Estudios Universitarios, Universidad Autónoma de Madrid, Calle La Salle, 10, 28036 Madrid, Spain; 3 Multidisciplinary Group on Pain Research and Management, Excellence Research Program URJC-Santander, Universidad Rey Juan Carlos, Alcorcón, Spain; 4 Departamento de medicina y cirugía, psicología, medicina preventiva y salud pública e inmunología microbiología médica, Universidad Rey Juan Carlos, Avda. Atenas s/n, 28922 Alcorcón, Spain; 5 Department of Physical Therapy, Occupational Therapy, Rehabilitation and Physical Medicine, Universidad Rey Juan Carlos, Avda. Atenas s/n, 28922 Alcorcón, Spain

**Keywords:** Temporomandibular disorders, Craniofacial disability, Neck disability, Headache, Chronic pain

## Abstract

**Background:**

The objective was to compare and correlate disability, pain intensity, the impact of headache on daily life and the fear of movement between subgroups of patients with chronic temporomandibular disorder (TMD).

**Methods:**

A cross-sectional study was conducted in patients diagnosed with chronic painful TMD. Patients were divided into: 1) joint pain (JP); 2) muscle pain (MP); and 3) mixed pain. The following measures were included: Craniomandibular pain and disability (Craniofacial pain and disability inventory), neck disability (Neck Dsiability Index), pain intensity (Visual Analogue Scale), impact of headache (Headache Impact Test 6) and kinesiophobia (Tampa Scale of Kinesiophobia-11).

**Results:**

A total of 154 patients were recruited. The mixed pain group showed significant differences compared with the JP group or MP group in neck disability (*p* < 0.001, *d* = 1.99; and *p* < 0.001, *d* = 1.17), craniomandibular pain and disability (*p* < 0.001, *d* = 1.34; and *p* < 0.001, *d* = 0.9, respectively), and impact of headache (*p* < 0.001, *d* = 1.91; and *p* < 0.001, *d* = 0.91, respectively). In addition, significant differences were observed between JP group and MP group for impact of headache (*p* < 0.001, *d* = 1.08). Neck disability was a significant covariate (37 % of variance) of craniomandibular pain and disability for the MP group (β = 0.62; *p* < 0.001). In the mixed chronic pain group, neck disability (β = 0.40; *p* < 0.001) and kinesiophobia (β = 0.30; *p* = 0.03) were significant covariate (33 % of variance) of craniomandibular pain and disability.

**Conclusion:**

Mixed chronic pain patients show greater craniomandibular and neck disability than patients diagnosed with chronic JP or MP. Neck disability predicted the variance of craniofacial pain and disability for patients with MP. Neck disability and kinesiophobia predicted the variance of craniofacial pain and disability for those with chronic mixed pain.

## Background

According to Medical Subject Headings, pain is considered chronic when it is an aching sensation that persists for more than a few months. It might or might not be associated with trauma or disease, and can persist after the initial injury has healed. Its localization, character and timing are vaguer than with acute pain.

In general, Craniofacial pain and disability, is a health problem that affects a large population. Chronic temporomandibular disorders (TMD) are included in this classification, with a high prevalence [1] and pain duration of more than 15 days per month, continuously or in episodes of at least 4 h [2] and for more than 3 months.

Chronic TMD can present persistent, recurrent or chronic pain associated with temporomandibular joint dysfunction and/or muscles involved in the masticatory system [3]. The etiology of chronic TMD is multifactorial and related to functional, structural and psychological factors [4–6]. TMD has been shown have an impact on both physical and psychological factors [1]. Its prevalence is estimated at between 3.7 and 12 %, and it is at least, twice more common in women than in men (2:1 to 9:1) [7, 8]. One of its most common clinical symptoms is pain, which can affect areas such as the ears, eyes and/or throat, frequently causing neck pain and headache [9]. Physical factors could be due to an inflammatory process, such as trauma, secondary synovitis, infection or irritation. Chronic TMD is also typically related to joint dysfunctions such as disc displacement with or without reduction [10]. TMD is classified according to international diagnostic criteria (DC/TMD) that separate the physical (Axis I) and psychological (Axis II) symptoms. Axis I includes, among others, joint pain (JP) disorders, muscle pain (MP) disorders and headaches attributed to TMD [11, 12]. Schiffman et al. recommended that future studies will allow for an improved taxonomic system based on signs and symptoms, and ultimately lead to a diagnostic system based on mechanism and etiology [13]. Therefore, investigate the etiological differences in TMD pains, it might be helpful to progress in this target.

TMD-related disability is one of the most important condition observed in chronic TMD [14]. Similarly, craniomandibular and neck disability have been associated with painful chronic TMD [15]. On th other hand, pain intensity and fear of jaw movements play an important role in the decision to seek care for orofacial pain and concretely, women with more fear of jaw movements were more likely to seek care [16].

Ciancaglini and Radaelli have shown that patients with chronic TMD are predisposed to headaches, thus it is of great importance to assess the impact of headache in these cases [17]. The relationship between chronic TMD and various headaches could be due to similarity in the pathophysiology of both diseases [18]. Although still unconfirmed, most studies blame trigeminal nucleus cervical modulation for the amplification of pain in this region. According to the literature, patients with chronic TMD are predisposed to develop a process of central sensitization [19–22]. Increased sensitization of the nociceptive receptors could affect the response of the afferent nerve fibers, causing central hyperexcitability of the neurons in the dorsal horn of the spinal cord, leading to plastic changes in spinal and/or supraspinal levels. These changes could lead to an alteration in the descending pathways of pain modulation [23], facilitating pain widespread and disability. It is also important to stress that patients with chronic pain suffer from significant fear of movement, which increases the rate of disability [24, 25].

The authors of this article, hypothesize that disability, pain intensity, the impact of headache on daily life and the fear of movement could be different between subgroups of patients with chronic TMD. Therefore, the primary aim of this study was to compare mandibular and neck disability and its association with craniofacial pain intensity, the impact of headache in daily life and fear of movement between subgroups of patients with chronic TMD.

## Methods

### Design

To improve the quality of our study, we used the Strengthening the Reporting of Observational Studies in Epidemiology (STROBE) international guidelines [26].

This is a comparative cross-sectional study. The research was approved by the ethics committee of La Paz University Hospital (LPUH code PI-1241), at which the study was developed between January and October 2014.

### Participants

Patients were recruited from the orofacial pain unit of LPUH. In the recruiting phase, each patient was assigned to one of the following groups according to their diagnosis, which was established by the DC/TMD: 1) chronic TMD with JP; 2) chronic TMD with MP; and 3) mixed chronic TMD [27]. All participants signed a consent informed.

The following were the exclusion criteria: 1) presentation of a systemic, rheumatic or central nervous system disease; 2) combined diagnosis of chronic migraine and chronic TMD; 3) fibromyalgia; 4) history of trauma or recent surgery on the head, face, neck or chest; 5) receiving physiotherapy at the time of evaluation; 6) younger than 18 years old; and 7) pregnancy. A total of 850 patients were excluded from the study due to the exclusion criteria.

### Variables

There were 5 variables considered, all of which were quantitative. First, the levels of craniofacial pain and disability were assessed using the Craniofacial Pain and Disability Inventory (CF-PDI), which has good psychometric properties. The CF-PDI consists of 21 items (range 0–63 points) based on two factors: pain and disability, and the functional status of the jaw [28]. The Minimal Detectable Change (MDC) was 7 points.

Second, neck disability was evaluated using the Neck Disability Index (NDI). The NDI comprises 10 items, of which only the first and sixth refer to pain, whereas the rest refer to activities in connection with that pain. Each item is scored from 0 (no disability) to 5 (total disability) and can earn a maximum of 50 points [29]. MDC and Minimal Important Change (MIC) on NDI (scale 0–50) were 8.4 and 3.5 points, respectively. Changes should exceed this MDC or MIC cut-off to be interpreted as relevant [30].

The third variable considered was the impact of headache on daily life. This variable was evaluated by the Headache Impact Test (HIT-6), which is made up of 6 items evaluating a headache’s impact on the patient’s quality of life. This test has been shown to be both reliable and valid [31]. The total score is obtained by adding the points for each item, for a minimum of 36 points and a maximum of 78. Scores above 60 points are considered as headaches with a severe impact on the life of the patient [31].

Fourth, the visual analog scale (VAS) was used to evaluate pain intensity. The VAS comprises a 100-mm horizontal line from 0 mm representing “no pain” to 100 mm representing “pain as bad as you can imagine”. The patient marked the line at the point they felt represented the pain intensity at the time, which was quantified by the assessor in mm. This scale has shown its reliability and validity for the measurement of pain intensity [32].

Finally, the level of fear of movement and re-injury was assessed using the short version of the Spanish validated Tampa Scale for Kinesiophobia (TSK-11). This scale consists of two factor models, called *Activity Avoidance* and *Harm* [33]. In respect of specific cut-off scores, a reduction of at least 4 points maximise the likelihood of correctly identifying an important reduction in fear of movement.

### Potential sources of bias

To avoid selection bias, established inclusion and exclusion criteria were defined to reduce differences between the study populations. To avoid classification bias, the patients received their medical diagnosis by a specialist in chronic TMD in order to be classified in the correct group. Another important bias could have been ingesting medication; to prevent this bias, the patients were reminded not to take any medication 24 h prior to measurement, apart from preventive medication. Finally, with the aim of preventing information bias, all the patients received comprehensive information about the study.

An independent researcher and specialist in chronic TMD was responsible for the diagnosis of all patients according to RDC/TMD [27]. A blinded evaluator to diagnosis of the patients, was an experienced physiotherapist expert in TDM.

### Sample size

To calculate the sample size, the G*Power 3.1 program developed at the University of Düsseldorf was used [34].

A power calculation was used to detect differences between groups in pain-related disability and psychological variables. Because detecting differences between groups was our primary interest, a F- Test was employed. The calculation used a size effect f of 0.25 (moderate), based on a pilot study with a sample of 18 subjects, obtaining 80 % statistical power (1-β error probability) with an α error level probability of 0.05, and suggested a sample size of 159 participants. Added, given that the second aim of this study proposed the creation of a regression analysis to assess the association between variables, it was necessary to expand the sample, considering that we used 4 predictors. To meet this goal, a minimum of 40 participants per group was required; given the regression analysis, the rule of 10 cases per variable was applied to obtain reasonably stable estimates for the regression coefficients [35].

### Statistical methods

All the data analyses were performed using SPSS for Windows, version 21.0 (SPSS Inc., Chicago, IL, USA). Descriptive statistics were generated for the sociodemographic, psychological and pain-related disability variables. The results are expressed as mean, standard deviation (SD), with 95 % confidence intervals (95 % CI). A normal distribution of the data was confirmed with the Kolmogorov-Smirnoff test. The weight, education level and chronicity measures did not meet the normal distribution. However, a normal distribution for all the variables was assumed according to the central limit theorem. This theorem states that the distribution of the average of a large number of independent variables (i.e., a large sample size is considered from 30 to 50 or more subjects) will be approximately normal regardless of the underlying distribution [36, 37].

A one-way ANOVA was used to analyze the group factor for pain-related disability and psychological variables (CF-PDI, NDI, HIT-6, VAS, TSK-11). Significant ANOVA findings were followed up with a post hoc test using the Bonferroni correction. Partial eta-squared (η^2^_p_) was calculated as a measure of effect size (strength of association) for each main effect and interaction in the ANOVAs, with 0.01–0.059 representing a small effect, 0.06–0.139 a medium effect and >0.14 a large effect [38]. Cohen’s *d* effect-sizes were calculated for multiple comparisons of the outcome variables. According to Cohen’s method, the magnitude of the effect was classified as small (0.20–0.49), medium (0.50–0.79), or large (0.8) [39].

The relationship between pain-related and psychological measures was examined using Pearson correlation coefficients. A Pearson correlation coefficient greater than 0.60 indicated a strong correlation, a coefficient between 0.30 and 0.60 indicated a moderate correlation and a coefficient below 0.30 indicated a low or very low correlation [40].

A multiple linear regression analysis was performed to estimate the strength of the associations between the results on craniofacial disability. Psychological and pain-related disability variables were used as predictors. Variance inflation factors (VIFs) were calculated to determine whether there were any multicollinearity issues in any of the 3 models. The strength of association was examined using regression coefficients (β), *P* values and adjusted *R* ^2^. Standardized beta coefficients were reported for each predictor variable included in the final reduced models to allow for direct comparison between the predictor variables in the regression model and the criterion variable being studied.

## Results

A total of 154 patients were recruited, of whom 57.11 % were men, with an average age of 45.19 (12.75) years (mean (SD)), with a weight of 66.81 (10.28) and a height of 1.64 (0.08). No statistically significant differences were found in age, weight and height between the groups for any of the values (*p* > 0.05). Duration of symptoms (in months with pain related-diagnoses), showed statistically significant differences when comparing mixed chronic TMD with JP (mean difference of 50.56 months; *p* < 0.001) and MP (mean difference of 37.56 months; *p* = 0.003). No differences were found in duration of symptoms between JP and MP in chronic TMD.

**Fig. 1:**
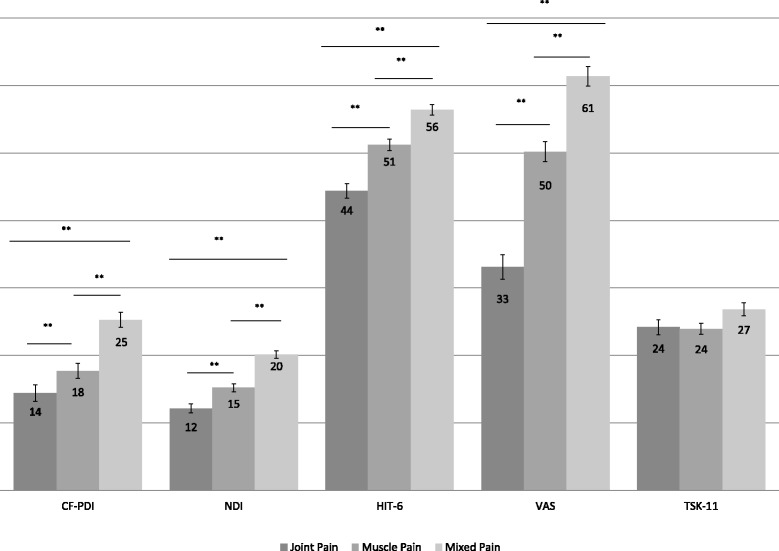
Comparison between groups. Mean and Standard Error. CF-*PDI* Craniofacial Pain and Disability Inventory, *NDI* Neck Disability Index, *HIT*-*6* Headache Impact Test, *VAS* Visual Analogic Scale, *TSK*-*11* Tampa Scale of Kinesiophobia. *p-value < 0.05. **p-value < 0.01

According to the diagnoses, 43 patients had chronic JP, 59 patients had chronic MP and 52 patients had mixed chronic pain. No differences were found between groups for sex (*p* > 0.05) and the women distribution within subgroups was 55.8 % in JP, 42.4 % in MP and 32.7 % in mixed group. Statistically significant differences were observed in the groups for craniofacial pain and disability (*F* = 21.87; *p* < 0.001; η^2^_p_ = 0.26), neck disability (*F* = 39.84; *p* < 0.001; η^2^_p_ = 0.36), impact of headache (*F* = 45.25; *p* < 0.001; η^2^_p_ = 0.40) and pain intensity (*F* = 75.07; *p* < 0.001; η^2^_p_ = 0.50). No differences were found between groups for kinesiophobia (*p* > 0.05) (Fig. 1).

The mixed pain group showed statistically significant differences compared with the JP group on the neck disability scale (*d* = 1.99; *p* < 0.001) or compared with patients diagnosed with MP (*d* = 1.17; *p* < 0.001). The mixed pain group showed statistically significant differences compared with the JP group on the craniofacial pain and disability scale (*d* = 1.34; *p* < 0.001) and compared with the patients diagnosed with MP (*d* = 0.9; *p* < 0.001). The JP group also showed statistically significant differences (*d* = 0.68; *p* = 0.002) in neck disability compared with the group diagnosed with chronic MP. Regarding the impact of headache, statistically significant differences were observed between all the groups for multiple comparisons when comparing chronic mixed pain with JP (*d* = 1.91; *p* < 0.001), mixed pain with MP (*d* = 0.91; *p* < 0.001) or JP with MP (*d* = 1.08; *p* < 0.001). There were no differences between the groups in terms of fear of movement or kinesiophobia (Table 1).

**Table 1:** Mean comparison between groups (Mean ± Standard Deviation)

Groups comparisons	Variable	Mean Difference	Interval Confidence 95 %	Cohen *d*
A) MP vs JP	CF-PDI	3.29	−0.74–7.33	0.39
B) MP vs Mixed		−7.55**	−11.39– −3.73	−0.90
C) JP vs Mixed		−10.85**	−15.00– −6.70	−1.34
A) MP vs JP	NDI	3.06**	0.96–5.17	0.68
B) MP vs Mixed		−4.92**	−6.96– −2.89	−1.17
C) JP vs Mixed		−7.95**	−10.19– −5.78	−1.99
A) MP vs JP	HIT-6	6.85**	3.71–9-98	1.08
B) MP vs Mixed		−5.17**	−8.12– −2.22	−0.91
C) JP vs Mixed		−12.02**	−15.08– −8.96	−1.91
A) MP vs JP	VAS	17.05**	11.62–22.49	1.47
B) MP vs Mixed		−11.16**	−16.31– −6.00	−1.02
C) JP vs Mixed		−28.21**	−33.80– −22-62	−2.53
A) MP vs JP	TSK-11	0.24	−3.48–3.01	−0.04
B) MP vs Mixed		−2.87	−6.02–0.27	−0.45
C) JP vs Mixed		−2.64	−6.04–0.76	−0.38

*MP* Muscle Pain, *JP* Joint Pain, CF-*PDI* Craniofacial Pain and Disability Inventory, *NDI* Neck Disability Index, *HIT*-*6* Headache Impact Test, *VAS* Visual Analogic Scale, *TSK*-*11* Tampa Scale of Kinesiophobia

*p-value < 0.05

**p-value < 0.01

### Correlation analysis (Table 2)

**Table 2:** Pearson’s correlation coefficients

	VAS	CF-PDI	NDI	TSK-11	HIT-6
Joint Pain					
VAS	1				
CF-PDI	0.404**	1			
NDI	−0.036	0.248	1		
TSK-11	0.048	0.221	0.086	1	
HIT-6	0.466**	0.443**	0.364*	0.177	1
Muscle Pain					
VAS	1				
CF-PDI	0.156	1			
NDI	0.307*	0.439**	1		
TSK-11	0.170	0.300*	0.446**	1	
HIT-6	0.311*	0.212	0.383**	0.254	1
Mixed Pains					
VAS	1				
CF-PDI	0.194	1			
NDI	0.154	0.535**	1		
TSK-11	0.307*	0.485**	0.460**	1	
HIT-6	0.119	0.306*	0.167	0.163	1

*JP* Joint Pain, *MP* Muscle Pain, CF-*PDI* Craniofacial Pain and Disability Inventory, *NDI* Neck Disability Index, *HIT*-*6* Headache Impact Test, *VAS* Visual Analogic Scale, *TSK*-*11* Tampa Scale of Kinesiophobia

*p-value < 0.05

**p-value < 0.01

Considering the Pearson correlation coefficient divided by group, the patients diagnosed with chronic MP showed moderate positive correlations between neck disability and craniofacial disability (*r* = 0.439; *p* = 0.001) and between kinesiophobia and neck disability (*r* = 0.446; *p* < 0.001). Regarding the chronic JP group, a moderate positive correlation was shown between craniofacial disability and pain intensity (*r* = 0.404; *p* = 0.007), between the impact of headache and pain intensity (*r* = 0.466; *p* = 0.002) and between the impact of headache and craniofacial disability (*r* = 0.443; *p* = 0.004).

Finally, moderate positive correlations were observed in the mixed chronic pain group between neck disability and craniofacial disability (*r* = 0.535; *p* < 0.001), between kinesiophobia and craniofacial disability (*r* = 0.485; *p* = 0.001) and between kinesiophobia and neck disability (*r* = 0.460; *p* = 0.001).

### Multiple linear regression

A multiple linear regression model using the variable CF-PDI as the criterion is shown in Table 3. Neck disability was a significant covariate (37 % of variance) of craniofacial pain and disability for the MP group (β = 0.62; *p* < 0.001). Neck disability (β = 0.40; *p* < 0.001) and kinesiophobia (β = 0.30; *p* = 0.03) were significant covariate (33 % of variance) of craniofacial pain and disability for the mixed chronic pain group. In addition, kinesiophobia (β = 0.34; *p* = 0.03) was a significant covariate (9 % of variance) of craniofacial pain and disability for the JP group.

**Table 3:** Multiple linear regression

Dependent variable: CF-PDI					
Group					
Joint Pain	Model R^2^ = 0.12	R^2^ Adjusted = 0.09	F = 4.97		
	Covariate	Regression Coefficient (B)	Standarized coefficent (β)	*p* value	VIF
	TSK-11	0.3	0.34	0.03	1.00
	Excluded Variables				
	VAS	-	−0.02	0.87	-
	NDI	-	0.27	0.08	-
	HIT-6	-	0.16	0.3	-
Muscle Pain	Model R^2^ = 0.39	R^2^ Adjusted = 0.37	F = 27.61		
	Covariate	Regression Coefficient (B)	Standarized coefficent (β)	*p* value	VIF
	NDI	0.83	0.62	0.001	1.00
	Excluded Variables				
	VAS	-	0.15	0.22	-
	TSK-11	-	0.06	0.65	-
	HIT-6	-	0.05	0.97	-
Mixed Pain	Model R^2^ = 0.36	R^2^ Adjusted = 0.33	F = 4.98		
	Covariate	Regression Coefficient (B)	Standarized coefficent (β)	*p* value	VIF
	NDI	0.84	0.40	0.001	1.27
	TSK-11	0.35	0.30	0.03	1.27
	Excluded Variables				
	VAS	-	0.47	0.71	-
	HIT-6	-	0.18	0.15	-

CF-*PDI* Craniofacial Pain and Disability Inventory, *NDI* Neck Disability Index, *TSK*-*11* Tampa Scale of Kinesiophobia, *HIT*-*6* Headache Impact Test, *VAS* Visual Analogical Scale, *VIF* Variance inflation factors

## Discussion

### Craniofacial and neck disability

In comparison with our study, Olivo et al. investigated women with chronic TMD, comparing MP and mixed pain groups with asymptomatic subjects. This study showed statistically significant differences between the MP group and the mixed pain and asymptomatic groups, but there was no difference between the other patient groups [41]. This result could be because 100 % of their sample was female and they only compared MP and combined chronic TMD groups with asymptomatic subjects.

A 2013 study of patients with chronic TMD, comparing with present data, did not find significant differences between subgroups with respect to jaw disability. A possible reason is the high percentage of patients they included in the chronic MP group (64.9 %), the use of other tools to assess disability (jaw disability check list) or the inclusion criteria of more than 6 months for a chronic condition [42].

On the other hand, supporting our study, recent research on various patients with orofacial pain, including chronic TMD, determined that significant differences were obtained in craniofacial disability only when compared with articular and muscle pain groups [28].

As for possible correlations between craniofacial disability and other variables, our study established moderate positive correlations between craniofacial disability, neck disability, impact of headache and pain intensity. However, we observed a low positive correlation between craniofacial disability and fear of movement. Similar to our results, numerous studies have found strong and very strong positive correlations between pain intensity and neck disability with respect to craniofacial disability [41, 43, 44].

La Touche et al. found a strong positive correlation between neck disability and craniofacial pain and disability [28].

Silveira et al. performed a study in which 20 women with a diagnosis of chronic TMD participated, although unlike our work, the patients were not classified into subgroups. Nevertheless, they observed a strong positive correlation between jaw disability and neck disability; thus, it is important to include the evaluation of neck disability in such patients [45].

Neck disability was a strong predictor of craniofacial pain and disability in the MP group and conformed to a prediction model of kinesiophobia for the mixed pain group. These results have clinical implications. Treatment needs to be focused on neck and craniofacial areas because the improvement of one could have an influence on the other [46, 47].

### Impact of headache on daily activities

In our study, we found that patients with mixed chronic pain had a higher headache impact (56.38 points) than the patients in the JP and MP groups; this score represents an important impact on the patients’ quality of life. In addition, the MP group (51.21 points) showed more headache impact than the JP group (44.37 points).

The results for the articular and muscle groups are similar to a recent study conducted for validation and development of a craniofacial disability questionnaire, in which they included patients with various types of chronic TMD and patients with primary headaches. They found a significant impact of 54.48 points [28]. Any increased sensitization of the nociceptive receptors could affect the response of the afferent nerve fibers, causing central hyperexcitability of the neurons in the dorsal horn of the spinal cord affecting trigeminal nucleus and facilitating headache symptoms.

In a recent university study to determine the annual prevalence of primary headaches, Souza-e-Silva y Rocha-Filho found a severe impact of headache on quality of life. These results are similar to those obtained in the mixed chronic TMD group in the present study; therefore, the impact of headache on quality of life could be similar for people who suffer from primary headaches and people who suffer from mixed chronic pain [48].

These results are supported by a recent study that found patients who have poorer results after receiving conservative treatment for pain, are those who had chronic TMD associated with headache [49]. Certainly, it was found a direct relationship between headache and chronic TMD. In a longitudinal study, patients with headache had 2.7 times greater probability of developing chronic TMD [50].

Perhaps, future studies could assess the impact of headache among cases with a diagnosed headache attributed to TMD.

### Kinesiophobia

In the present study, all the subgroups of patients presented a similar level of kinesiophobia, and although there were no statistically significant differences in this measure between various groups, they had a similar level as a recent study on other musculoskeletal pain [51].

Few studies have researched the relationship between kinesiophobia and chronic TMD. However, in a 2010 study that evaluated the validity and reliability of the TSK-11 adapted to chronic TMD, Visscher et al. found that patients with chronic TMD who have more functional problems related to the jaw joint suffered a greater degree of fear of movement. Also, fear of movement is strongly related to mechanical jaw problems, such as sounds or blocks [52]. That situation could explain why in our study, the JP group had no differences with MP and/or mixed pain groups in TSK-11. Moreover, fear of movement is the only variable that no differences have been showed.

The score obtained in these groups with chronic TMD is similar to that found in patients with chronic widespread pain (28 points) and lower than that of patients with low back pain (33 points) [53]. The score is similar to that found in another study on patients with chronic TMD, which used a questionnaire adapted for kinesiophobia [52].

Strong evidence suggests that kinesiophobia is a predictor of disability in patients with various types of chronic pain, including TMD [44, 53, 54]. The present study found a positive moderate correlation between fear of movement and neck disability in the chronic mixed pain and MP TMD groups, but not in the articular chronic TMD group. Also, a positive moderate correlation was found between the mixed chronic TMD group and the level of kinesiophobia and the craniofacial disability index.

Recent studies support the idea that chronic TMD is a multifactorial disorder, in which psychological factors play an important role in the onset and development of the pathology. Finally, it is clinically important to emphasize that the vast majority of patients with chronic TMD have a mixed diagnosis (85 %) [55].

Kinesiophobia was a predictor of craniofacial pain and disability in the JP group and conformed to a prediction model of neck disability for the mixed pain group. Thus, neck disability and kinesiophobia could be influencing craniomandibular pain and disability. Again, this situation has important clinical implications in the evaluation and treatment of these patients.

### Limitations

Several limitations should be taken into account in the present study. It was not possible to compare our results with longitudinal studies because there is a lack of these types of studies in the literature. First, it is remarkable that 57 % of the study-population was men since almost all clinical samples women outnumber men (3–5 as many women as men); this may be due to a bias associated to consecutive bootstrapping used in this study. These results could have been different with a usual sex distribution. Our study did not collect physical variables such as craniomandibular range of motion, which could provide new and interesting information. Another important limitation is the lack of a Spanish version of the TSK-11 for TDM and it is recommended to developed it in further studies according translation and adaptation process [56]. The scale used in this study was a short version of a general kinesiophobia scale validated in the Spanish language. Also, although all the recruited patients had a chronic evolution, there were differences between the mixed pain group and the JP and MP groups. Therefore, caution should be used when interpreting these results due to the differences in chronicity possibly interfering with the results. Authors tried to balance all groups, however and due to issues related with bootstrap and prevalence, the JP group was less participants (43 versus 59 for MP and 52 for mixed pain). Besides, the Graded Chronic Pain Scale could have been used to increase the possibilities for comparisons with other questionnaires and future studies should take in account this regard.

This study provides important information regarding psychosocial factors that appear in patients with chronic TMD, which could be disability predictors. Thus, we suggest considering these factors as likely predictors in the evaluation and treatment of these patients.

## Conclusion

Patients with mixed chronic pain diagnosis show greater craniomandibular and neck disability than patients with a diagnosis of chronic JP or MP. In addition, patients with mixed chronic pain show greater headache impact than the chronic JP or MP groups. Neck disability predicted a 37 % variance of craniofacial pain and disability in MP. Neck disability and kinesiophobia predicted a 33 % variance of craniofacial pain and disability for patients with mixed chronic pain.

## Abbreviations

CF-PDI: Craniofacial pain and disability inventory
HIT-6: Headache impact test
JP: Joint pain
LPUH: La Paz University Hospital
MP: Muscle pain
NDI: Neck disability index
RDC/TMD: Research diagnostic criteria for temporomandibular disorders
STROBE: Strengthening the Reporting of Observational Studies in Epidemiology
TMD: Temporomandibular disorders
TSK-11: Tampa scale for kinesiophobia
VAS: Visual analog scale
VIFs: Variance inflation factors
